# Transformerless Ultrasonic Ranging System with the Feature of Intrinsic Safety for Explosive Environment

**DOI:** 10.3390/s18124397

**Published:** 2018-12-12

**Authors:** Yu Wang, Yuheng Qiao, Hongjuan Zhang, Yan Gao, Ming Zhang, Heng Tan, Dong Wang, Baoquan Jin

**Affiliations:** 1 Key Laboratory of Advanced Transducers and Intelligent Control Systems (Ministry of Education and Shanxi Province), College of Physics and Optoelectronics, Taiyuan University of Technology, Taiyuan 030024, China; wangyu@tyut.edu.cn (Y.W.); wangdong@tyut.edu.cn (D.W.); 2 College of Electrical and Power Engineering, Taiyuan University of Technology, Taiyuan 030024, China; qiaoyuheng0266@link.tyut.edu.cn (Y.Q.); zhanghongjuan@tyut.edu.cn (H.Z.); gaoyan@tyut.edu.cn (Y.G.); tanheng0207@link.tyut.edu.cn (H.T.); 3 Center of Nanosciences and of Nanotechnologies, University of Paris-Saclay, 91405 Orsay, France; ming.zhang@u-psud.fr

**Keywords:** ultrasound transducer, ultrasonic ranging, intrinsic safety, transformerless driving

## Abstract

The transformer used in the conventional ultrasonic ranging system could provide a huge instantaneous driving voltage for the generation of ultrasonic wave, which leads to the safety problem in the explosive mixture. This paper proposes a transformerless ultrasonic ranging system powered by the intrinsically safe power source and analog switches. The analysis of intrinsic characteristics of ultrasonic driving circuit is realized in normal and fault conditions. The echo-processing circuit combined with LIN bus technology is further adopted in order to improve the system stability. After the analysis of the timing diagram of ranging instruction, the evaluation experiments of ranging accuracy and ranging stability are completed. The results show that the system can realize reliable bidirectional communication between the LIN master node circuit and the ultrasonic transceiver unit, which realizes the transformerless driving. The system can realize the distance measurement within the range of 250–2700 mm, and the measurement error is less than 30 mm. The measurement fluctuation is less than 10 mm, which provides a new solution for the ultrasonic ranging system in the potentially explosive atmosphere.

## 1. Introduction

Due to the narrow working space in the industrial field, many special function vehicles have hidden hazards in the process of reversing, posing a huge threat to safety production [1]. In order to solve these kinds of problems, the ultrasonic detection technology is thus widely used for monitoring and early warning of safety distances because of its simple structure and fast response [2,3,4,5,6], and could be gradually seen in the potentially explosive atmospheres of gases and vapors.

The traditional ultrasonic ranging system driven by the transformer has the disadvantages of large volume and high cost. In this situation, a large driving voltage is required to drive the primary and secondary coils, and a high energy storage is generated while ensuring the driving performance [7]. These effects could be dangerous in explosive atmospheres, because the ultrasound has been considered to be an ignition source in the international safety regulations such as EN 1127-1 [8]. The ignition point of methane–air mixture is about 650 °C. In Reference [9], it is mentioned that the temperature required to ignite combustibles by hot surface methods is higher (about hundreds of degrees). However, this condition is far from being met during the operation of ultrasonic ranging system. Therefore, this paper focuses on the ignition caused by electric sparks. Moreover, Reference [10] even suggested a new safety threshold value of 170 dB for ultrasound coupled directly to gaseous atmospheres. Actually, in normal or fault conditions, when the energy released in the equipment circuit is higher than the minimum ignition energy of the material in the environment, it may ignite the surrounding dust or gas [11,12]. For exemple, the large driving voltage in the driving circuit of ultrasound transducer is an important hazards, because the electric spark may be generated if the system is in fault conditions, such as short circuits.

Therefore, in the environment of blasting dust and explosive gas, electrical and electronic equipment must be designed to meet the requirements of safe production [13,14]. For the explosion-proof environment, the explosion-proof design of electrical and electronic equipment often uses the oil immersion type, sand filling type, flameproof enclosure, intrinsically safe type, and encapsulation type to separate the safe area from the non-safe area. Among them, the intrinsically safe equipments are widely used, because they eliminate the explosion-proof casing and other complicated structures, and have many advantages such as light weight, small size, low cost, and best safety.

In this paper, we focus on the intrinsic safety design of ultrasonic ranging circuit, and the effect of ignition source ultrasound is not considered here. The effective driving circuit of the ultrasonic transducer under a small voltage is proposed to improve the energy conversion efficiency. The acoustic impedance matching and electrical impedance matching [15,16,17,18] are also used to achieve this goal. At the same time, considering the complicated situation in the potentially explosive atmospheres of gases and vapors, the stability of the entire ranging system is improved by introducing the Local Interconnect Networks (LIN) bus which is widely used in the automobile network [19,20]. In summary, the new approach in this paper is that a transformerless ultrasonic ranging system is proposed and the intrinsic characteristics of ultrasonic driving circuit are realized in normal and fault conditions. The echo-processing circuit combined with LIN bus technology is further adopted in order to improve the system stability.

## 2. Intrinsically Safe Driving of an Ultrasonic Transducer

### 2.1. Driving Principle of an Ultrasonic Transducer

In the ultrasonic ranging system, piezoelectric ceramic transducers are often used to realize real-time acquisition of position information. The work of piezoelectric ceramic transducers is based on the inverse piezoelectric effect of piezoelectric crystals, which convert electrical energy into mechanical energy and then into acoustic energy. Under the action of the ultrasonic transducer system, the piezoelectric wafer is resonated by the signal as its own natural frequency, and the resonant plate that is attached there to emit ultrasonic waves.

**Figure 1:**
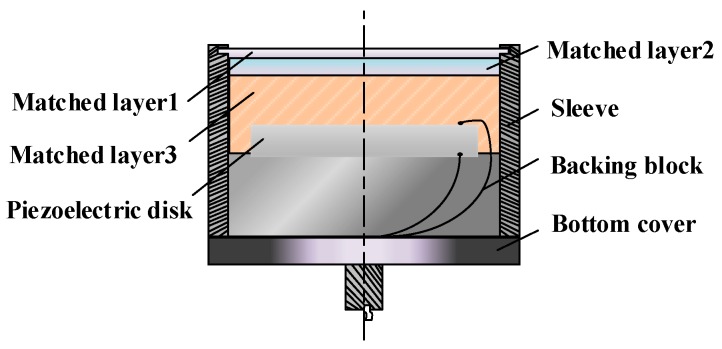
The internal structure of an ultrasonic transducer.

The overall conversion efficiency of an ultrasonic transducer system mainly depends on power transmission efficiency, mechanical energy transmission efficiency, and electromechanical conversion efficiency. An important indicator affecting the conversion efficiency of mechanical energy and electrical energy is the electromechanical coupling coefficient of piezoelectric crystal materials. This coefficient represents the ratio of the mechanical energy converted by the piezoelectric crystal to the total energy input by the inverse piezoelectric effect in the ultrasonic driving section, which is closely related to the shape of the element, the crystal composition material, and the cutting direction. Since piezoelectric crystals in ultrasonic ranging systems are mostly developed, such as piezoelectric ceramic transducer (PZT)-based piezoelectric ceramics which have been developed, a space for efficiency improvement of the piezoelectric material itself, especially the electromechanical coupling coefficient, has been very limited. In order to meet the requirements of intrinsically safe driving, it is necessary to optimize the energy matching and mechanical energy matching transmission efficiency to realize the driving in the ultrasonic transducer at a small voltage. The internal structure of the ultrasonic transducer is shown in Figure 1.

### 2.2. Acoustic Impedance Matching of the Ultrasonic Transducer

**Figure 2:**
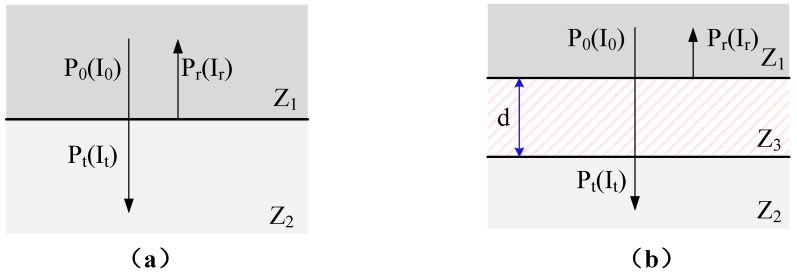
The schematic of ultrasonic vertical incidence. (**a**) Acoustic wave incident directly, (**b**) acoustic wave incident through the matched layer.

The sound waves of the ultrasonic transducer used for ultrasonic ranging are transfered from a solid environment to a gaseous environment. When the sound waves pass through the two media interfaces, they will generate reflected waves and transmitted waves. As shown in Figure 2a, when sound wave is vertically incident from medium 1 to medium 2, the sound transmittance *t* and the sound intensity transmittance *T* satisfy the following conditions [21]:

(1) $$t=\frac{P_{t}}{P_{0}}=\frac{2Z_{2}}{Z_{1}+Z_{2}}$$

(2) $$T=\frac{I_{t}}{I_{0}}=\frac{4Z_{1}Z_{2}}{(Z_{1}+Z_{2}{)}^{2}}$$

where *Z*_1_ and *Z*_2_ are the acoustic impedances of media 1 and 2, respectively; *P*_0_ and *P_t_* are the incident and transmitted sound pressures, respectively; and *I*_0_ and *I_t_* are the incident and projected sound intensities, respectively. The acoustic impedance *Z* is satisfied as following:

(3) Z=ρc

where ρ is the medium density and *c* is the wave velocity in the medium. It can be seen from the Equation (3) that the acoustic transmittance at the interface is related to the material itself. In actual use, if the emitting surface is made of aluminum, the sound intensity transmission *T* is:

(4) $$T=\frac{I_{t}}{I_{0}}=\frac{4Z_{1}Z_{2}}{(Z_{1}+Z_{2}{)}^{2}}=0.0094\%$$

The problem of too low transmission intensity at the time of direct incidence is shown in Figure 2b. Improvement is made by adding a matching layer, medium 3, between media 1 and 2. When the thickness d of medium 3 satisfies the following equation, there is a maximum sound intensity transmittance *T_max_*. The thickness d of medium 3 and the maximum sound intensity transmittance *T_max_* can be written as:

(5) $$d=\frac{\gamma }{4}2n-1,n=1,2,3\ldots$$

(6) $$T_{max}=\frac{4Z_{1}Z_{3}^{2}Z_{2}}{{\left(Z_{1}Z_{2}+Z_{3}^{2}\right)}^{2}}$$

where *γ* is the wavelength of the sound wave in medium 3, and $Z_{3}$ is the acoustic impedance of medium 3. When the acoustic impedance $Z_{3}$ of medium 3 satisfies the equation: $Z_{3}=\sqrt{Z_{1}Z_{2}}$, $T_{max}$ takes a value of 1, which means the sound signal does not reflect at all. Therefore, it can be known that the sound transmission efficiency can be improved by reasonably selecting the matching material and the matching layer thickness, thereby improving the overall energy transmission efficiency of the overall driving system.

**Figure 3:**
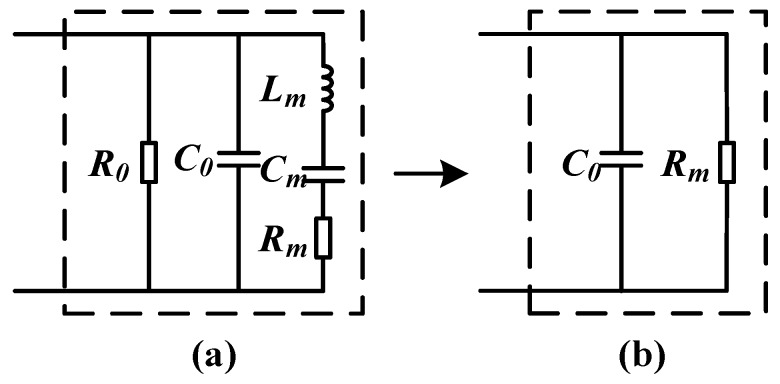
Equivalent circuit structure of the ultrasonic transducer: (**a**) equivalent circuit diagram of the transducer before resonance, (**b**) equivalent circuit diagram of the transducer after resonance.

The mechanical vibration characteristics of ultrasonic piezoelectric transducers can also be expressed in terms of electrical quantities. In order to analyze the load impedance of the transducer, it is necessary to construct a transducer equivalent circuit by matching the appropriate inductor or capacitor to improve the energy conversion efficiency of the transducer. The equivalent circuit of the ultrasonic transducer is shown in Figure 3.

Figure 3a is the equivalent circuit diagram of the ultrasonic transducer before resonance, where *C*_0_ is the static capacitance of the ultrasonic transducer due to clamping, and *R*_0_ is the dielectric resistance of the ultrasonic transducer. Since *R*_0_ is considered to be infinite, the influence is generally ignored. $L_{m}$, $C_{m}$ and $R_{m}$ are the dynamic inductance, capacitance, and resistance of the transducer, respectively. When the frequency of transducer driving pulse is equal to the resonant frequency $f_{s}$ of the ultrasonic transducer,$\;L_{m}$ and *C_m_* undergo series resonance. The series branch is pure resistance *R_m_*, the equivalent circuit of the transducer is shown in Figure 3b, $R_{m}$ and *C*_0_ are connected in parallel, and the impedance is capacitive. The resonant frequency $f_{s}$ of the ultrasonic transducer is described as:

(7) $$f_{s}=\frac{1}{\sqrt{L_{m}C_{m}}}$$

### 2.3. Design of the Intrinsically Safe Ultrasonic Driving Circuit

The core of the ultrasonic transducer system is a layered resonance structure based on piezoelectric materials, which shows the characteristics of capacitive load. Therefore, it is necessary to adopt electrical impedance matching method to improve the output power of the signal source to the transducer during power transmission. In order to realize the transmission of ultrasonic waves, it is necessary to design a pulse driving circuit to generate an electric pulse having the same resonance frequency as that of the ultrasonic transducer and having a large driving voltage, and to drive the ultrasonic transducer to vibrate after matching the network, for the purpose of transmitting ultrasonic waves.

**Figure 4:**
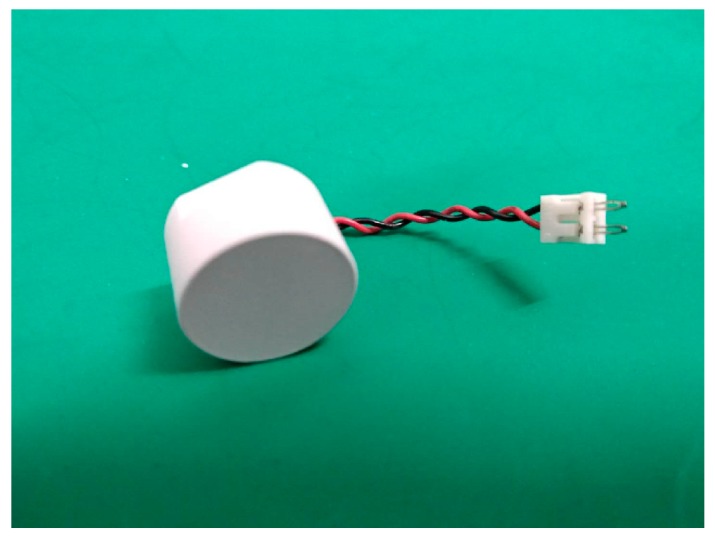
Physical map of the ultrasonic transducer.

The ultrasonic transducer used in this scheme is an anti-drip-type transceiver-integrated ultrasonic probe (model: MA58MF14-0N), which is shown in Figure 4, and its electrical performance parameters are listed in Table 1.

**Figure 5:**
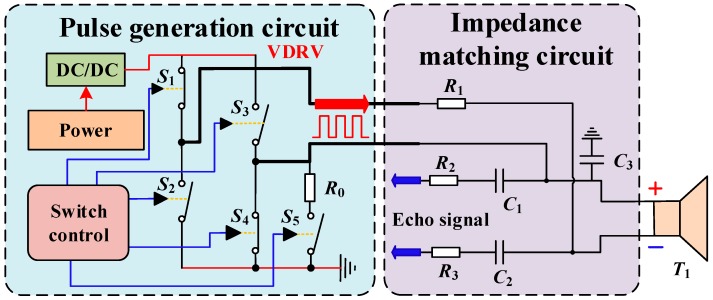
Intrinsically safe ultrasonic driving circuit.

In order to meet the intrinsically safe driving requirements, a transformerless ultrasonic driving circuit is designed and shown in Figure 5. The circuit consists of the impedance matching and the pulse generation. *R*_1_ and *C*_3_ are the matching resistor and the matching capacitor of the driving circuit, respectively. The echo-receiving capacitors *C*_1_ and *C*_2_ and the echo-receiving resistors *R*_2_ and *R*_3_ are chosen carefully in order to complete the reception of the echo signal.

The system is powered by an intrinsically safe power supply (18 V) and produces the required driving voltage *V_DRV_* (12 V) via a direct current (DC)/DC converter. The pulse generation circuit is composed of four analog switches and controllers, i.e., *S*_1_, *S*_2_, *S*_3_, and *S*_4_. When the positive pulse is driven, the switches *S*_2_ and *S*_3_ are turned on, and *S*_1_ and *S*_4_ are turned off. At this time, the positive voltage of the transducer is *V_DRV_*, the negative terminal is 0; in the negative pulse driving state, the switches *S*_1_ and *S*_4_ are turned on, and *S*_2_ and *S*_3_ are turned off. At this time, the negative voltage of the transducer is *V_DRV_* and the positive terminal is 0. When the controller controls the switch to turn on and off with the resonant frequency *f_s_* of the transducer, a square wave of amplitude *V_DRV_* and frequency *f_s_* can be generated at both ends of the transducer, thereby ensuring normal operation of the transducer. After the end of a set of 16 pulses, the controller controls *S*_0_ to close, and the damping *R*_0_ is connected to the circuit to reduce the ultrasonic “tailing” caused by mechanical inertial vibration, for the purpose of reducing the dead zone.

### 2.4. Analysis of Intrinsic Characteristics of the Ultrasonic Driving Circuit

#### 2.4.1. Analysis of Intrinsic Characteristics of Circuit In Normal Conditions

**Figure 6:**
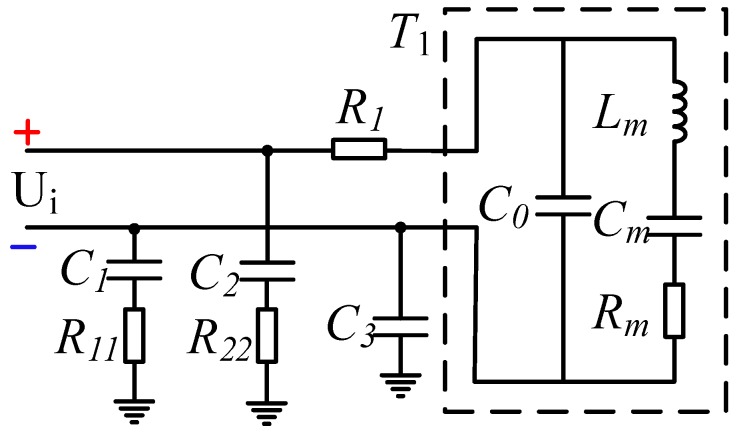
The circuit model of driving system.

In the driving circuit of the ultrasonic ranging circuit, the energy storage component has a large energy storage, so it is difficult to meet the intrinsic safety requirements. Therefore, the capacitor energy storage is the first consideration of the design, and the energy storage of the capacitor is calculated as follows:

(8) $$\mathrm{Wc}=\frac{1}{2}CU^{2}$$

where *U* is the voltage across the capacitor *C*, and in order to meet the intrinsic safety performance requirements of the ultrasonic ranging device, combined with the equivalent circuit model of the ultrasonic transducer and the transformerless ultrasonic driving circuit in Figure 3 and Figure 5, respectively, the circuit model of the driving system shown in Figure 6 is obtained, and the description of each parameter is shown in Table 2. *R*_1_ is the matching resistor and *C*_3_ is the matching capacitor. *C*_1_ and *C*_2_ are the echo-receiving capacitors. These four designations of the components have the same designations, as those in Figure 5. *C*_0_ is the static capacitance. *L_m_*, *C_m_*, and *R_m_* are the dynamic inductance, capacitance, and resistance of the transducer, respectively. These four designations of the components have the same designations as those in Figure 3. *R*_11_ and *R*_22_ are equivalent resistances between the echo-receiving terminal and the ground.

**Figure 7:**
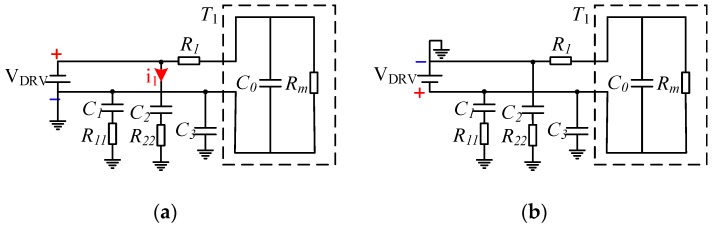
The equivalent circuit model of different driving states (**a**) for positive pulse driving and (**b**) for negative pulse driving.

The driving pulse is obtained by the switch at a frequency *f* and the voltage source *V_DRV_*. When the driving pulse frequency *f* is equal to the resonance frequency of the ultrasonic transducer, *L_m_* and *C_m_* undergo series resonance. The series branch is a pure resistor *R_m_*. In the ultrasonic-distance-measured device, the 16-pulse driving method is usually used, and the voltage between the two ends of the driver (+, −) is obtained as follows:

(9) $$\mathrm{Ui}+\left(n\right)=\frac{V_{DRV}}{2}({\left(-1\right)}^{n+1}+1)$$

(10) $$\mathrm{Ui}-\left(n\right)=\frac{V_{DRV}}{2}({\left(-1\right)}^{n}+1)$$

(11) $$\mathrm{Ui}\left(n\right)=V_{DRV}{\left(-1\right)}^{n+1}$$

where *n* (*n* = 1, 2, 3, …, 32) is a half-pulse ordinal. Then, during the positive and negative pulses, the equivalent circuit of the driving system is shown in Figure 7. The descriptions of the components in Figure 7 are shown in Table 2 and these element parameters in Figure 7 have the same meanings as those in Figure 6.

As shown in Figure 7a, during positive pulse driving, *C*_1_ and *C*_3_ are short-circuited to the ground. If *C*_2_ is stored, its energy is

(12) $$W_{C_{02}}=\frac{1}{2}CU^{2}=0.5\times 470\times 10^{-12}\times 12^{2}=33.84\times 10^{-9}J=33.84\;nJ$$

The actual positive pulse duration is *t_p_*, which is given by:

(13) $$t_{p}=\frac{1}{2}\times \frac{1}{f}=0.5\times \frac{1}{58\times 10^{3}}=8.62\times 10^{-6}s=8.62\;\mu s$$

During this period, the path formed by *C*_2_ and *R*_22_ satisfies:

(14) $$R_{22}\times C_{2}\times \frac{dU_{C_{2}}}{\mathrm{dt}}+U_{C_{2}}=V_{DRV}$$

Then before the end of the positive pulse,

(15) $$U_{C_{2}}=V_{DRV}\left(1-e^{\frac{-t}{R_{22}\times C_{2}}}\right)=12\times \left(1-e^{\frac{-8.62\times 10^{-6}}{100\times 10^{3}\times 470\times 10^{-12}}}\right)=2.0112\;V$$

Then, the actual energy is:

(16) $$W_{C_{2}}=\frac{1}{2}CU^{2}=0.5\times 470\times 10^{-12}\times 2.0112^{2}=33.84\times 10^{-9}J=0.95\;nJ$$

According to the national and international standards, in the potentially explosive atmospheres of gases and vapors with class I (methane–air), the minimum ignition energy *W_min_* = 0.28 mJ [22,23], according to the above results $W_{C_{2}}\ll W_{C_{02}}\ll W_{min}$, so the selection of capacitor *C*_2_ meets the intrinsic safety requirements.

**Figure 8:**
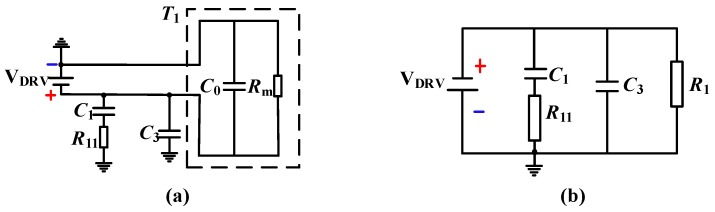
System equivalent circuit diagrams for the conditions of (**a**) short circuit at *R*_1_ and (**b**) short circuit at *T*_1_.

Similarly, as shown in Figure 8b, during the negative pulse driving, *C*_2_ is short-circuited to the ground. If *C*_1_ and *C*_3_ are stored, their respective energies are given by:

(17) $$W_{C_{01}}=\frac{1}{2}CU^{2}=0.5\times 470\times 10^{-12}\times 12^{2}=33.84\times 10^{-9}J=33.84\;nJ$$

(18) $$W_{C_{03}}=\frac{1}{2}CU^{2}=0.5\times 220\times 10^{-12}\times 12^{2}=15.84\times 10^{-9}J=15.84\;nJ$$

According to the above calculation results, $W_{C_{01}}\ll W_{min}$, and $W_{C_{03}}\ll W_{min}$, so the capacitors *C*_1_ and *C*_3_ are selected to meet the intrinsic safety requirements.

#### 2.4.2. Analysis of Intrinsic Characteristics of Circuit in Fault Conditions

Considering that short-circuit faults may be relatively difficult to happen in the internal circuit of the ultrasonic transducer, the conditions of the short circuit of the matching resistor *R*_1_ and the short-circuit of the transducer *T*_1_ are analyzed. The equivalent circuit is shown in Figure 8. The descriptions of the components in Figure 8 are shown in Table 2 and these element parameters in Figure 8 have the same meanings as those in Figure 6.

When the short circuit of *R*_1_ occurs, the negative pulse driving mode is more dangerous than the positive pulse driving. Therefore, as shown in Figure 8a, the system is a capacitive circuit, and the safety factor of the power supply voltage should be 1.5 according to the book by Zhang and Li [24]. Then, the corrected voltage is 1.5*V_DRV_* = 18 V, and the maximum equivalent capacitance of the system is $C_{e_{1}}$ = *C*_1_ + *C*_3_ + *C*_0_ = 2090 pF. It is checked according to the minimum ignition curve (*C* + 0 Ω) of the class I capacitive circuit, at 20 V (greater than 18 V), and the corresponding capacitance value is *C*_*min*1_, (20 μF < *C*_*min*1_ < 30 μF), that is, $C_{e_{1}}\ll C_{min1}$, so the intrinsic safety requirement is also satisfied at this time.

Similarly, as shown in Figure 8b, when the short circuit of the transducer *T*_1_ occurs, the system is a capacitive circuit. According to the standard, the safety factor of the power supply voltage should be 1.5, and the corrected voltage is 1.5*V_DRV_* = 18 V. The equivalent capacitance is $C_{e_{2}}$ = *C*_1_ + *C*_3_ = 690 pF, so it is checked according to the minimum ignition curve (*C* + 40 Ω) of the class I capacitive circuit. At 20 V (greater than 18 V), the corresponding capacitance value is *C*_*min*2_ ($C_{min2}\gg 100\;\mu F$), that is, $C_{e_{2}}\ll C_{min2}$, so it also meets the requirements of intrinsic safety.

## 3. Design of Ultrasonic Echo Signal Processing Based on LIN Bus Technology

### 3.1. LIN Bus and Its Network Structure

**Figure 9:**
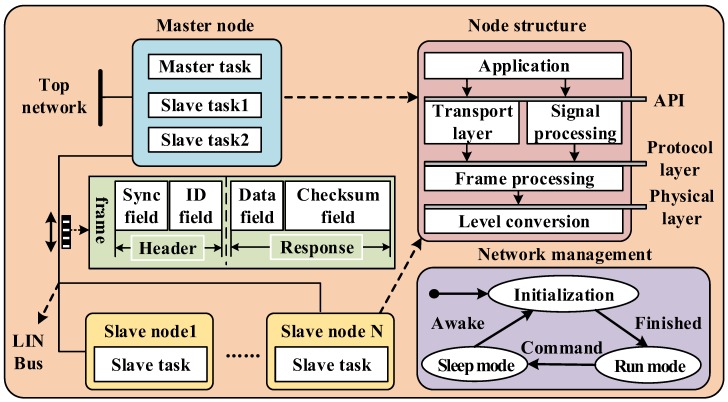
The diagram of Local Interconnect Network (LIN) network and node composition.

In order to realize the communication and parameter setting of the microcontroller in the ultrasonic transceiver unit, the scheme adopts the LIN bus transceiver chip TJA1020. LIN is a serial communication protocol based on the Universal Asynchronous Receiver/Transmitter (UART)/Serial Communication Interface (SCI) interface, which can be used in various fields such as automobiles and home appliances. As shown in Figure 9, the LIN network as a whole adopts a single bus mode, and the network will enter the running mode after the initialization process is completed; when the sleep command is received or the bus is silent for 4–10 s, the mode will be going into sleep mode, and the network will enter initialization mode by wake-up signals or internal causes. The function of the LIN network is implemented by different nodes such as the master node and the slaves. The node application layer transmits signals and messages downwards through the protocol layer and the physical layer, and it can expand up to 16 nodes in an LIN network. The master node in the LIN network can not only control the slaves in the network, detect and adjust the bus status, but also communicate with the upper layer network (such as Controller Area Network (CAN)), which has both master and slave tasks, while the slave node only contains slave tasks. 

The communication message frame format on the LIN bus is fixed, and the bus communication is controlled by the master node. After the master node passes the interval signal, the master node sends the synchronization field and the identifier field as the header of the message, and the slave returns the data field and the checksum field response host. Due to the physical layer limitation, the LIN bus transmission bit rate is up to 20 kbps, and the following conditions should be met during communication:

(19) $$T_{\mathrm{Header}}=34\cdot T_{\mathrm{bit}}$$

(20) $$T_{\mathrm{Response}}=(10\cdot N_{\mathrm{Data}}+10)\cdot T_{\mathrm{bit}}$$

where *T*_Header_ is the nominal transmission time of the header, *T*_bit_ is the time required for the primary node to transmit 1 bit of data, *T*_Response_ is the response-rated transmission time, and *N*_Data_ is the number of bytes contained in the data field. At the same time, in order to ensure the transmission, both *T*_Header_ and the *T*_Response_ need to leave a certain margin, and the maximum transmission time is 1.4 times the rated transmission time.

### 3.2. Design of the Ultrasonic Echo Signal Processing Circuit

**Figure 10:**
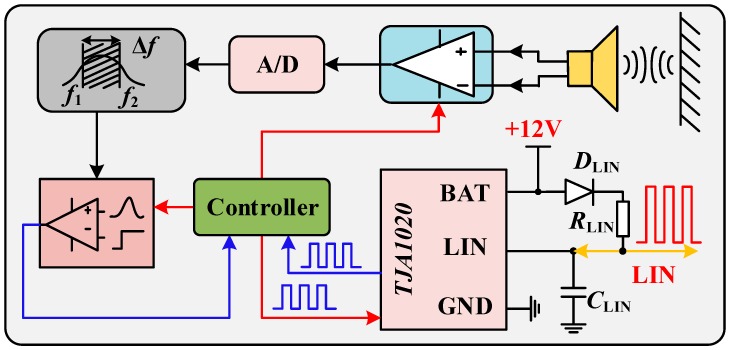
Echo-processing circuit.

The system echo-processing flow is shown in Figure 10. When the ultrasonic wave is affected by the obstacle and the echo signal is generated, the piezoelectric crystal in the ultrasonic transducer is forced to vibrate to convert the sound signal into a weak electrical signal. The weak electrical signal is amplified by the programmable amplification module and converted into a digital voltage signal through Analog to Digital (A/D) conversion. The digital filter is used to filter out clutter outside 58 kHz to reduce its interference to the system. The filtered signal passes through the echo threshold comparison module. When it is greater than the given value, it is the effective timing termination point. In the process of echo-processing, since the calculation of the obstacle distance time point is generated by voltage threshold comparison, the setting of the reference value for echo comparison is crucial.

In order to improve the stability and anti-interference ability of the echo-processing system, the reverse current diode *D*_LIN_ and the LIN matching resistor *R*_LIN_ are connected in series between the LIN and Battery supply (BAT) pins of the module. The LIN node capacitor *C*_LIN_ is used to control the voltage fluctuation in the LIN. Moreover, the TJA1020-enabled host application mode is used to receive the master controller’s instructions and return echo information, as shown in Figure 10. The main controller communicate with the echo controller through LIN to control parameters such as amplification gain and echo comparison threshold. The echo controller returns the threshold comparison result and current status information.

### 3.3. LIN Bus Communication Performance Optimization

In order to ensure the bidirectional operation of the LIN communication to receive the signal fed back by the ultrasonic transceiver unit, the LIN bus voltage should satisfy the threshold condition.

At the dominant level,

(21) $$V_{\mathrm{LIN}\_L\_\mathrm{MAX}}<0.4\cdot V_{\mathrm{BAT}}=4.8\;V$$

At the recessive level,

(22) $$V_{\mathrm{LIN}\_H\_\mathrm{MIN}}<0.6\cdot V_{\mathrm{BAT}}=7.2\;V$$

where *V*_LIN_L_MAX_ is the highest voltage at a low level in LIN bus; *V*_LIN_H_MIN_ is the minimum voltage at a high level in LIN bus; *V*_BAT_ is Chip BAT terminal voltage and equal to 12 V, and thus the size of *R*_LIN_ needs to be adjusted during the actual process to change the driving capacity of the circuit, so as to meet the requirements in the potentially explosive atmospheres of gases and vapors and implement effective data communication.

## 4. Realization of the Transformerless Ultrasonic Ranging System

### 4.1. System Principle Overview

**Figure 11:**
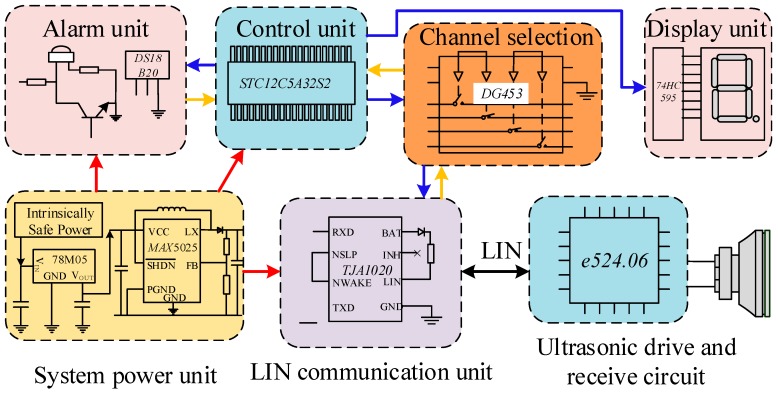
Block diagram of the ultrasonic ranging system.

The intrinsically safe transformerless ultrasonic ranging system is shown in Figure 11, and is mainly composed of a power supply unit, a control unit, a channel selection unit, an LIN communication unit, an ultrasonic transceiver unit, an alarm and calibration unit, and a display unit. The system uses STC12C5A32S2 microcontroller as the core of the control unit. The power supply unit is supplied with 18 V voltage from the intrinsically safe power supply (model: CSTI-I). The DC/DC conversion chip 78M05 provides the 5 V voltage required by the system and the MAX5025 generates 12 V voltage to ensure LIN communication. The channel selection unit realizes switching of the output signal of the control unit, and the LIN communication unit completes conversion of the Transistor-Transistor Logic (TTL) level and the LIN level to realize state control and data transmission of the ultrasonic transceiver unit with the e524.06 chip as the core.

### 4.2. Design of e524.06 Chip Peripheral Circuit

**Figure 12:**
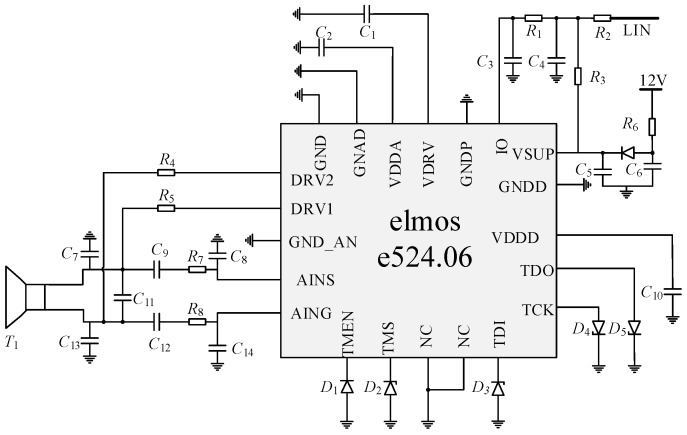
Ultrasonic transmitting and receiving circuit diagram.

The potentially explosive atmospheres of gases and vapors are complex and therefore put high demands on the circuit so the system uses the ultrasonic-distance-measuring chip e524.06 which was produced by an elmo company, and designed its peripheral circuit (as shown in Figure 12) to achieve transformerless driving and echo-processing of the ultrasonic transducer. The chip is internally composed of a power module, a transducer driving module, an echo-receiving and -processing module, a clock module, a Joint Test Action Group (JTAG) interface module, a controller module, an Electrically Erasable Programmable Read Only Memory (EEPROM) module, and an Input/Output (I/O) interface module. By modifying the EEPROM configuration information in the chip, not only the adjustment of the ultrasonic transducer driving voltage and frequency, but also the working state of the transducer can be changed, and the static and dynamic modes can be selected to generate the threshold. When static generation is selected, the relevant registers are configured to realize the setting of key parameters, such as linear interpolation, voltage offset and scale factor, to adjust the ultrasonic echo-processing effect to improve the system measurement accuracy.

### 4.3. Instruction and Ranging Timing of e524.06 Chip

**Figure 13:**
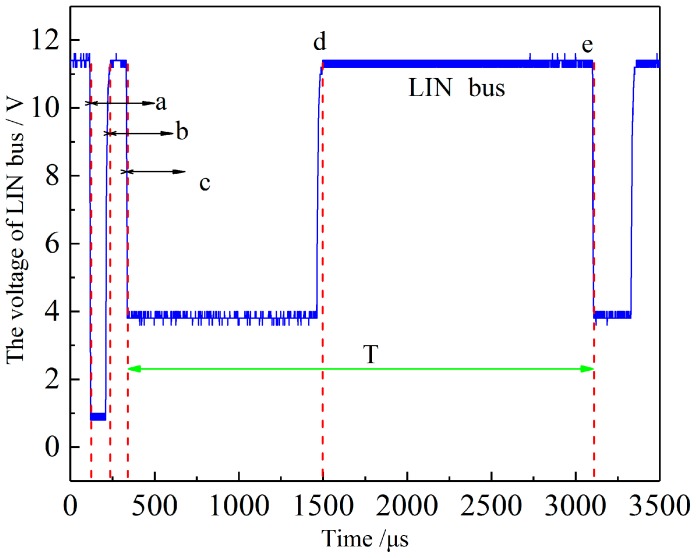
Timing diagram of the ranging command.

Writing specific instruction steps are required to realize communication between the microprocessor and e524.06 chip and other functions such as 16-pulse driving wave transmission and EEPROM programming. On the basis of completing the hardware design and compiling the signal path of the ultrasonic ranging system, the calculation of actual distance data is completed according to distance order sequence in Figure 13. The real-time voltage of LIN bus in the ranging process is shown in Figure 13. The system sends ranging instructions and waits for the respond of ultrasonic transceiver between a and b (a range of valid time intervals is 77.6–154 μs), e524.06 chip in the ultrasonic transceiver evaluates the environmental noise in the measurement process between c and d, and the length of time T is the time used for ultrasonic transmission during a single measurement of the system.

Software flow design of the whole ultrasonic ranging system can be realized according to the ranging distance sequence above. The controller issues a distance measurement command (the dominant level between 77.6 and 154 μs) after completing the system initialization, and starts the hardware count function, which calculates ultrasonic wave spreading time according to the difference between the counts of the two falling edges and system command cycle, with the help of programmable counter array (PCA) edge capture function of single-chip microcomputer (STC12C32S2).

### 4.4. Software Flow of the System Ranging Process

**Figure 14:**
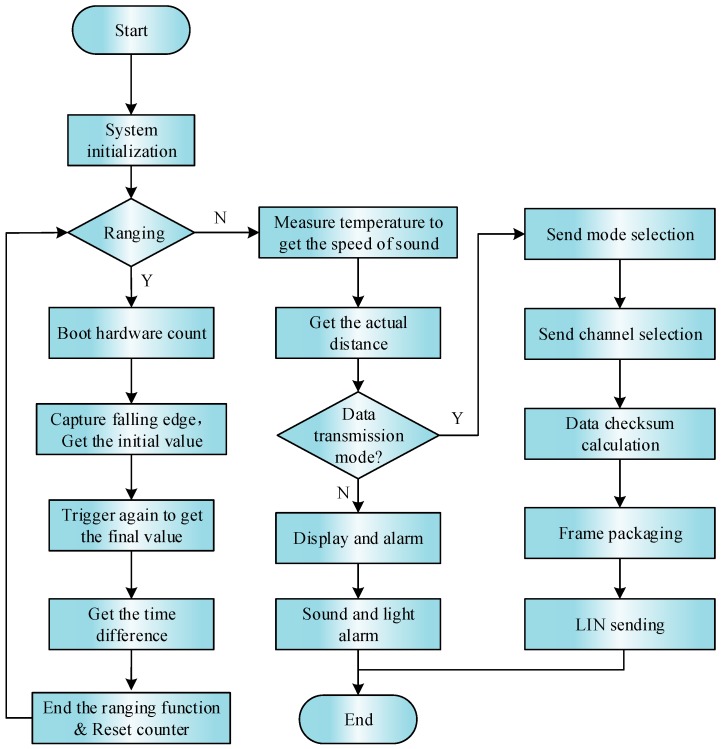
The diagram of system ranging process.

The working flow chart of the ranging system is shown in Figure 14. The system waits for the falling edge of c time (waiting for interrupt) from time b. When it enters the interrupt program, the system takes out the current timing value and uses it as the starting point of echo time. The system waits for the next falling edge trigger (e time) after processing the above, taking the current count value in the interrupt program as the end point of the echo time, clears the counter and related variables, and ends the ranging.

When the ranging is over, the system enters the communication and display module. Firstly, it turns off the capture function of the PCA module, uses the DS18b20 to get the current temperature, and further obtains the sound speed after calibration, and then calculates the current distance based on this. When data transmission is required, the system selects different transmission modes (control mode or acquisition mode), selects channels (single channel or quad channels), and then performs data checksum calculation. On this basis, it is packaged into data frames and sent via the LIN bus. When data transmission is not required, the real-time distance display is directly performed and an audible and visual alarm is performed according to the defined safety value.

## 5. Ultrasonic Ranging Experiment and Analysis

The level conversion and ranging test system are built to verify the analysis of ultrasonic driving system and LIN bus level part and evaluate the overall function and safety of the system.

### 5.1. Analysis of LIN-Bus-Matching Resistance Experiment

**Figure 15:**
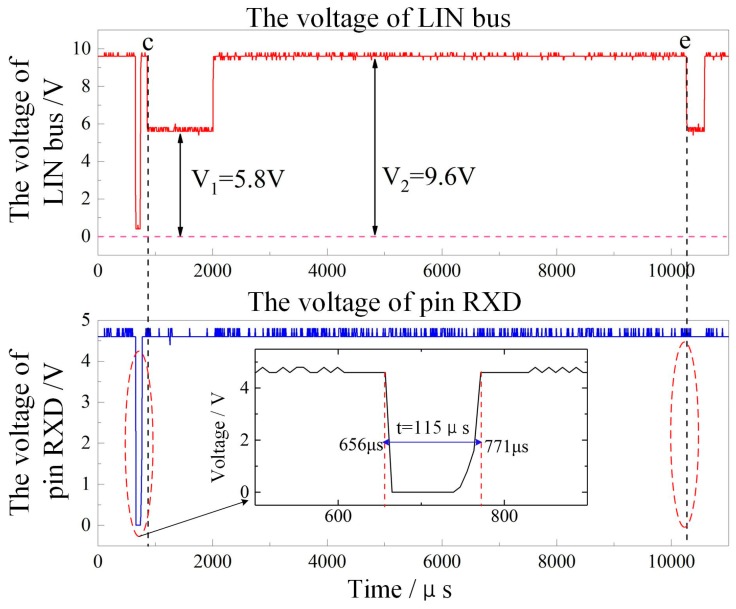
Diagram of the level on LIN communication (*R*_LIN_ = 1 kΩ).

As shown in Figure 15, it is noted that the controlling unit can realize the transmission of ultrasonic ranging instruction when the master node circuit *R*_LIN_ is 1 kΩ during the experiment. However, the controlling unit cannot receive the effective response of the ultrasonic transceiver, which is shown in Figure 15.

The two curves in Figure 15 are the level changes of the LIN bus and the receive data (RXD) port of the TJA1020 chip when the resistance *R*_LIN_ in Figure 10 is 1 kΩ, and the controller performs the ranging instruction (before c). The RXD terminal voltage varies with the LIN bus voltage (the dominant level is 115 μs), but at the time c and e, although the ultrasonic transceiver unit replies to the ranging information and the LIN bus generates a level change, the RXD terminal does not. The corresponding reaction is due to the fact that the “low” level of the LIN bus reaches 5.8 V, which exceeds the decision threshold of the TJA1020 for the highest level of the low level, so the hardware circuit needs to be improved.

Table 3 shows the communication level results when the *R*_LIN_ takes the values of 1 kΩ, 2 kΩ, and 3.5 kΩ. At this time, the ranging command transmission (123 μs, and 128 μs at the dominant level) can be realized. At point c and point e, as the voltage of the LIN bus drops, the voltage of RXD also changes. The corresponding low-level potentials of the LIN bus are 4.0 V and 3.2 V, respectively, in line with the LIN bus low level. The specification, therefore, produces a low level on the RXD side. The experiment also proves that when *R*_LIN_ are 2 kΩ and 3.5 kΩ, the circuit can complete normal communication and data interaction, and realize effective communication between the ultrasonic transceiver unit and the controlling unit.

### 5.2. Analysis of Ultrasonic Ranging Instructions and Driving Wave Experiments

**Figure 16:**
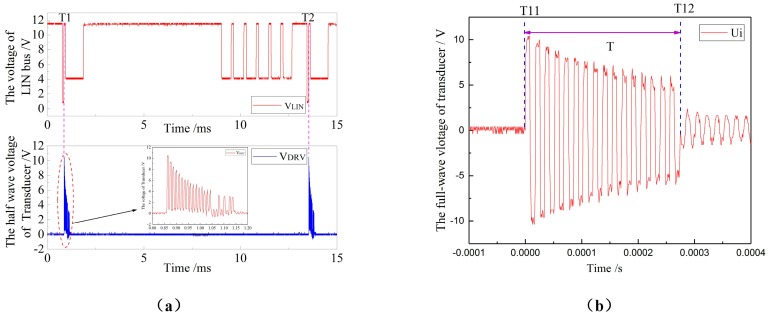
The diagram of ranging command and transducer voltage: (**a**) the voltage of LIN bus and half-wave voltage of transducer, and (**b**) the full-wave voltage of transducer.

In order to verify the validity of the ranging command of the communication control system composed of the system microprocessor and TJA2010 for the ultrasonic driving system, the results shown in Figure 16 can be obtained by monitoring the LIN command level and the voltage across the transducer. As shown in Figure 16a, at the time *T*_1_ and *T*_2_ after the ranging command is issued, a square wave is generated immediately at both ends of the transducer to drive the transducer. As shown in Figure 16b, a total of 16 sets of pulses are generated during the period *T*_11_–*T*_12_ of driving the transducer, and the frequency *f* is written as:

(23) $$f=\frac{n}{T_{12}-T_{11}}=\frac{16}{2.74599\times 10^{-4}-\left(-1.40043\right)\times 10^{-6}}=57.97\;kHz$$

### 5.3. Analysis of the System Measuring Range

**Figure 17:**
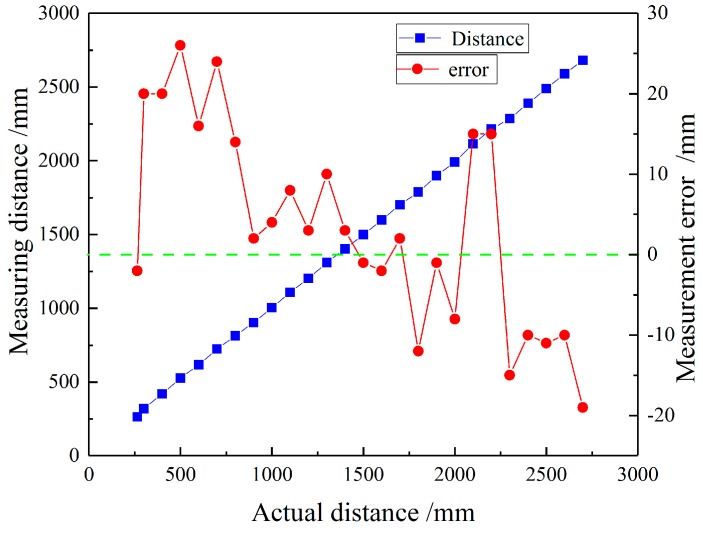
Diagram of the system’s measuring rang and error.

In the laboratory environment, a 30 cm × 30 cm × 5 mm iron plate and a 3-m-long guide rail and bracket were used to build a mining ultrasonic ranging experimental platform to measure the range and accuracy of the system. The distance measurement in the range from 240 to 2800 mm was performed in steps of 100 mm, and the results obtained are shown in Figure 17. The experiment proves that the system can measure the distance measurement within 250 mm to 2700 mm, and the measurement error increases when the measured distance is closer (within 800 mm) and farther (outside 2100 mm). When the distance is medium (900 mm–2000 mm), the error is smaller and the overall system error is less than 3 cm.

At the same time, in order to determine the maximum and minimum values of the system measurement, the experiment was carried out in steps of 5 mm in the range of 230–260 mm and 2650–2750 mm, and 30 mm was the maximum allowable error limit. The experiment results show that the error would be larger if the distance is less than 245 mm and more than 2705 mm, thus determining the overall measuring range of the system from 250 mm to 2700 mm.

### 5.4. Analysis of System Measuring Stability

**Figure 18:**
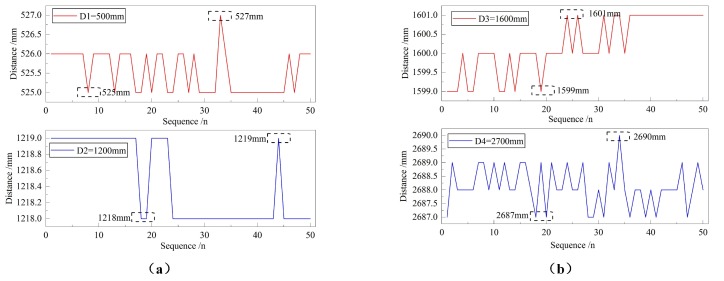
Diagram of system’s measuring stability test: (**a**) the distances of 500 mm and 1200 mm, and (**b**) the distances of 1600 mm and 2700 mm.

Considering that the system will make larger errors due to other factors such as the external environment in a single measurement, it is necessary to count the multiple measurement results of the system at the same distance to evaluate the stability of the system. Figure 18 shows the measurement results obtained after 50 times of measurements of the system at 500 mm, 1200 mm, 1600 mm and 2700 mm.

The experimental results show that when the obstacle distances are 500 mm, 1200 mm, 1600 mm and 2700 mm, the measurement fluctuations of the ultrasonic ranging system are 2 mm, 1 mm, 2 mm and 3 mm, respectively, with the fluctuation of less than 1 cm. At the same time, the maximum absolute value of measurement errors are 27 mm, 19 mm, 1 mm and 13 mm, respectively. The measurement at 1600 mm is more accurate, which is consistent with the moderate distance and small error experimental results in the measurement range experiment. The experimental result proves that the overall system error is less than 3 cm.

## 6. Conclusions

Based on the study of ultrasonic transmission path, this paper selects the ultrasonic transducer. The transformerless ultrasonic driving circuit was designed by calculating the energy of the critical energy storage components in normal and fault conditions. The parameter setting for implementing the wave-processing circuit based on LIN bus technology was completed, and the transmission scheme of the comparison result was demonstrated. Finally, the above-mentioned ultrasonic transceiver scheme was verified by the e524.06 chip, and the ultrasonic ranging system without a transformer was built to verify the ranging performance. The experiment proves that the ultrasonic ranging system can realize distance measurement in the range of 250–2700 mm with an error of less than 30 mm and a measurement fluctuation of less than 10 mm, which provides an effective method for improving the anti-interference ability of the mining ultrasonic ranging system.

## Figures and Tables

**Table 1:** Electrical parameters of ultrasonic transducer.

Nominal Frequency *f_s_* (kHz)	Static Capacitance *C*_0_ (pF)	Aftershock Time *T* (ms)	Maximum Input Voltage *V*_max_ (Vpp)
58 ± 1.5	1400 ± 20%	1.4	120

**Table 2:** Circuit parameters of the driving system.

**Driving** **Voltage U_i_ (V)**	**Matching Resistance *R*_1_ (Ω)**	**Matching Capacitance** ***C*_3_ (pF)**	**Echo-Receiving Capacitance** ***C*_2_ (pF)**
±12	100	220	470
**Equivalent Resistance** ***R*_11_ (kΩ)**	**Equivalent Resistance** ***R*_22_ (kΩ)**	**Static Capacitance** ***C*_0_ (pF)**	**Echo-Receiving Capacitance** ***C*_1_ (pF)**
100	100	1400	470

**Table 3:** Communication capability test result of variable resistances.

LIN-Matching Resistor *R*_LIN_ (kΩ)	LIN High Level *V*_2_ (V)	LIN LowLevel *V*_1_ (V)	Command Time *t* (μs)	RX Effectiveness
1	9.6	5.8	115	Invalid
2	8.6	4.0	123	Valid
3.5	7.8	3.2	128	Valid

## References

[1] Dash A.K., Bhattcharjee R.M., Paul P.S., Tikader M. (2015). Study and Analysis of Accidents Due to Wheeled Trackless Transportation Machinery in Indian Coal Mines—Identification of Gap in Current Investigation System. Procedia Earth Planet. Sci..

[2] Kumar S., Furuhashi H. (2017). Long-range measurement system using ultrasonic range sensor with high-power transmitter array in air. Ultrasonics.

[3] Yao Z., Hong L., Cheng L. (2016). Improvement of measurement distance in multi-channel ultrasonic ranging systems through adaptive chaotic pulse position width modulation excitation sequences. Insight Non-Destr. Test. Cond. Monit..

[4] Mu W.Y., Zhang G.P., Huang Y.M., Yang X.G., Liu H.Y., Yan W. (2016). Omni-Directional Scanning Localization Method of a Mobile Robot Based on Ultrasonic Sensors. Sensors.

[5] Gabriel G. (2011). Indoor Pedestrian Navigation Using Foot-Mounted IMU and Portable Ultrasound Range Sensors. Sensors.

[6] Yao Y., Ju X., Lu J., Men B. (2017). Acoustic Emission and Echo Signal Compensation Techniques Applied to an Ultrasonic Logging-While-Drilling Caliper. Sensors.

[7] Zhang H., Wang Y., Zhang X., Wang D., Jin B. (2016). Design and Performance Analysis of an Intrinsically Safe Ultrasonic Ranging Sensor. Sensors.

[8] (2011). CEN: Explosive Atmospheres e Explosion Prevention and Protection Part 1: Basic Conceptsand Methodology.

[9] Davis S., Kelly S., Somandepalli V. (2010). Hot Surface Ignition of Performance Fuels. Fire Technol..

[10] Simon L.H., Wilkens V., Beyer M. (2015). Safety-related conclusions for the application of ultrasound in explosive atmospheres. J. Loss Prev. Process. Ind..

[11] Addai E.K., Gabel D., Krause U. (2016). Experimental investigations of the minimum ignition energy and the minimum ignition temperature of inert and combustible dust cloud mixtures. J. Hazard. Mater..

[12] Zhang Q., Li W., Zhang S. (2011). Effects of spark duration on minimum ignition energy for methane/air mixture. Process. Saf. Prog..

[13] Li Y., Zhu H. (2018). A simple optimization method for the design of a lightweight, explosion-proof housing for a coal mine rescue robot. J. Braz. Soc. Mech. Sci. Eng..

[14] Solheim F., Arntzen B.J., Eckhoff R.K. (2012). Effect of rusting and mechanical damage of gap surfaces on efficiency of flame gaps in flameproof electrical apparatus. Process. Saf. Environ. Prot..

[15] Jahdali R.A., Wu Y. (2016). High transmission acoustic focusing by impedance-matched acoustic meta-surfaces. Appl. Phys. Lett..

[16] Toda M., Thompson M. (2010). Novel multi-layer polymer-metal structures for use in ultrasonic transducer impedance matching and backing absorber applications. IEEE Trans. Ultrason. Ferroelectr. Freq. Control.

[17] Kazys R.J., Sliteris R., Sestoke J. (2017). Air-Coupled Ultrasonic Receivers with High Electromechanical Coupling PMN-32%PT Strip-Like Piezoelectric Elements. Sensors.

[18] Kim H., Priya S., Stephanou H., Uchino K. (2007). Consideration of impedance matching techniques for efficient piezoelectric energy harvesting. IEEE Trans. Ultrason. Ferroelectr. Freq. Control.

[19] Terçariol W.L., Ferreira E.C., Dias J.A.S. (2012). Analogue control of the slew-rate in LIN bus digital transitions using translinear circuits. Analog Integr. Circuits Signal Process..

[20] Fang H., Han J., Wei Z. (2011). Modeling Method of Automotive Body CAN/LIN Nets Application Protocol Based on Object-oriented Colored Petri Net. Chin. J. Mech. Eng..

[21] Wan S., Jia M., Li L., Duan Y., Sun Y., Zhang W., Zheng X., Gao J., Song Y., Shi S. (2017). Ultrasonic Testing Technology and Application.

[22] Zhang B. (1981). Safety Spark Circuit.

[23] (2006). IEC/SC31G: Explosive Atmospheres-Part 11: Equipment Protection by Intrinsic Safety “i”.

[24] Zhang Y., Li W. (1991). Intrinsically Safe Circuit Design.

